# Antepartum stillbirth rates during the COVID‐19 pandemic in Austria: A population‐based study

**DOI:** 10.1002/ijgo.13989

**Published:** 2021-10-31

**Authors:** Dana A. Muin, Sabrina Neururer, Veronica Falcone, Karin Windsperger, Hanns Helmer, Hermann Leitner, Herbert Kiss, Alex Farr

**Affiliations:** ^1^ Department of Obstetrics and Gynecology Division of Feto‐maternal Medicine Medical University of Vienna Vienna Austria; ^2^ Department of Clinical Epidemiology Tyrolean Federal Institute for Integrated Care Tirol Kliniken GmbH Innsbruck Austria

**Keywords:** antepartum stillbirth, coronavirus disease 2019, epidemiology, fetal death, perinatal mortality

## Abstract

**Background:**

The coronavirus disease 2019 (COVID‐19) pandemic caused by severe acute respiratory syndrome coronavirus 2 has had dramatic effects on the pregnant population worldwide, increasing the risk of adverse perinatal outcomes.

**Objective:**

To assess the incidence of antepartum stillbirth (aSB) during the COVID‐19 pandemic in Austria.

**Methods:**

We collected epidemiological data from the Austrian Birth Registry and compared the rate of aSB (i.e., fetal death at or after 24^+0^ gestational weeks) during the pandemic period (March–December 2020) and in the respective pre‐pandemic months (2015–2019).

**Results:**

In total, 65 660 pregnancies were included, of which 171 resulted in aSB at 33.7 ± 4.8 gestational weeks. During the pandemic, the aSB rate increased from 2.49‰ to 2.60‰ (*P* = 0.601), in contrast to the significant decline in preterm deliveries at or before 37 gestational weeks from 0.61‰ to 0.56‰ (relative risk [RR] 0.93; 95% confidence interval [CI] 0.91–0.96; *P* < 0.001). During the first lockdown, the aSB rate significantly increased from 2.38‰ to 3.52‰ (*P* = 0.021), yielding an adjusted odds ratio of 1.57 (95% CI 1.08–2.27; *P* = 0.018). The event of aSB during the COVID‐19 pandemic was strongly related with increased fetal weight and maternal obesity.

**Conclusion:**

In Austria, there has been an overall increase in the incidence of aSB during the pandemic with a significant peak during the first lockdown.

## 1 INTRODUCTION

In March 2020, the World Health Organization declared coronavirus disease 2019 (COVID‐19)—caused by the the severe acute respiratory syndrome coronavirus 2 (SARS‐CoV‐2)—a global pandemic^1^ and ever since, the disease has had profound direct and indirect effects on the pregnant population worldwide. On the one hand, the direct effects of SARS‐CoV‐2 have caused a wide spectrum of symptoms in pregnant women in the case of infection, leading to a mild to severe maternal disease with increased risk for pulmonary disease and need for intensive care.^2^, ^3^ On the other hand, placental infections threaten fetal well‐being, contributing to both placental dysfunction and vertical transmission to the fetus.^4^

The indirect effect of the pandemic may hypothetically involve the individual's adoption of poor lifestyle habits due to social distancing, lockdowns, and redundancy, fostering unfavorable behaviors, such as smoking, high‐caloric intake, and becoming more sedentary.^5^ Also health‐seeking behavior rapidly decreased, resulting in less frequent clinical check‐ups. Lastly, closures and reductions in the public healthcare system with disruptions to follow‐up visits and regular obstetric monitoring significantly impacted the management of both low‐ and high‐risk pregnancies.^6^

We hypothesized that the restrictions in healthcare services and changes in prenatal care in Austria during the pandemic have negatively impacted perinatal outcomes, as reflected by the prevalence of antepartum stillbirth. We, therefore, conducted a population‐based study to assess the antepartum stillbirth rate—defined as fetal death at or after 24^+0^ gestational weeks, during the pandemic (March–December 2020) and in the pre‐pandemic era (matched months 2015–2019).

## 2 MATERIALS AND METHODS

### 2.1 Data collection and study design

**FIGURE 1:**
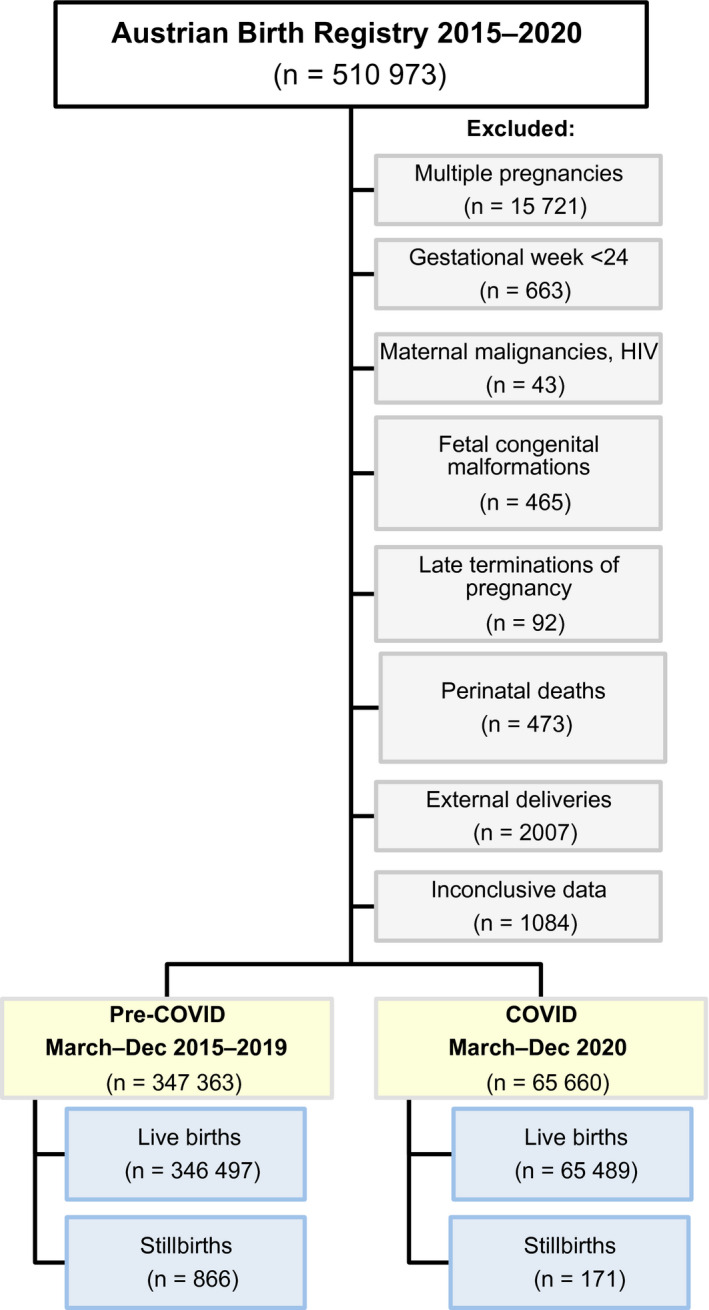
Flowchart on the selection of the study population from the Austrian Birth Registry between 2015 and 2020

The Austrian Birth Registry prospectively collects maternal demographic and perinatal data from the obstetrical database viewpoint (General Electric Company) of all maternity units in Austria, including registered home deliveries. For this study, we retrieved data from the Austrian Birth Registry during two distinct time periods: from March 16, 2020 to April 13, 2020 (reflecting the first lockdown phase in Austria), and from March 1, 2020 to December 31, 2020 (reflecting the pandemic era in Austria). Data were compared with the analogue periods between 2015 and 2019. We included all singleton live and stillborn deliveries at or after 24^+0^ gestational weeks. We excluded multiple pregnancies, intrapartum and perinatal deaths, and stillborn fetuses following late terminations of pregnancy and those with congenital anomalies (Figure 1). After a data check for integrity and consistency, all patient data were de‐identified and the database was frozen before analysis.

### 2.2 Definitions

In Austria, the government issued three lockdown phases to reduce the spread of SARS‐CoV‐2. The first lockdown was undertaken from March 16 to April 13, 2020 (i.e.,, 27 days), the second lockdown was from November 17 to December 6, 2020 (i.e., 19 days), and the third lockdown was from December 26, 2020 to February 7, 2021 (i.e., 41 days). As the first lockdown was the most tight and severe phase in this country, our analyses refer to this phase only.

### 2.3 Statistical analysis

Categorical data are presented as absolute (*n*) and relative (%) frequencies. We compared categorical data with the χ^2^ test, and continuous data with an unpaired *t* test. Log‐binomial regression and logistic regression were performed to assess risk ratios (RR) and odds ratios (OR). All ratios and difference measures are accompanied by a 95% confidence interval (CI). A two‐sided *P* value less than 0.05 represents statistical significance. The analyses were performed using STATA 16 (StataCorp LLC, College Station, TX, USA).

### 2.4 Ethical permission

The study was approved by the Ethics Committee of the Medical University of Vienna (Registration number 1637/2020) and complied with the principles as outlined in the Declaration of Helsinki and Good Clinical Practice guidelines. Participants' written consent was not required as per the Austrian Federal Act (Protection of Personal Data Regulation, §46, Paragraph 1; 2000).

### 2.5 Dissemination to participants and related patient and public communities

Patients and the public were not involved in the design, conduct, reporting, interpretation, and dissemination of the results of this study.

## 3 RESULTS

### 3.1 Baseline population characteristics

After consideration of the inclusion and exclusion criteria (Figure 1), during the pandemic between March and December 2020, a total of 65 660 singleton deliveries were registered in Austria at a mean ± standard deviation gestational age of 39.0 ± 1.7 weeks, of which 33 831 (51.5%) newborns were male and 31 805 (48.4%) were female (0.1% unreported sex; *n* = 24).

In the pre‐pandemic era between March and December 2015–2019, the total study population consisted of 347 363 deliveries of singletons at a mean gestational age of 39.0 ± 1.8 weeks, of which 178 533 (51.4%) newborns were male and 168 720 (48.6%) were female (0.1% of not reported sex, *n* = 110). Mean maternal age was 30.5 ± 5.3 years at the time of delivery, with a mean body mass index (BMI; calculated as weight in kilograms divided by the square of height in meters) of 23.9 ± 4.8 kg/m^2^.

During the pandemic and in comparison with the preceding years, the total pregnant female population was older (30.7 ± 5.3 years; *P* < 0.001), had a higher BMI (24.3 ± 5.3; *P* < 0.001), and a higher parity (25.8% versus 22.8%; *P* < 0.001). Likewise, mean newborn weight and length were higher during the pandemic than in the previous years (3364 ± 546 g vs. 2247 ± 521 g and 50.9 ± 3.3 cm vs. 50.7 ± 2.7 cm, respectively; *P* < 0.001).

### 3.2 Antepartum stillbirth rate during the COVID‐19 pandemic in Austria

The rate of preterm delivery at or before 37 gestational weeks decreased from 6.1% to 5.6% (RR 0.93; 95% CI 0.90–0.96) during the pandemic compared with the pre‐pandemic era (*P *< 0.001), the stillbirth rate increased from 2.49‰ to 2.60‰ (RR 1.1; 95% CI 1.06–1.17; *P *= 0.601; Table 1).

**TABLE 1:** Risk for stillbirth and preterm birth in Austria during the pandemic period (March to December 2020) in comparison with the same time period of the preceding years

	Events (per 1000 births)				Odds ratio (95% confidence interval)			
	Pre‐COVID‐19		COVID‐19					
	Births in March to December 2015–2019		Births in March to December 2020		Unadjusted	*P* value	Adjusted	*P* value
	(*n* = 347 363)		(*n* = 65 660)					
	*n*	‰	*n*	‰				
Stillbirth	866	2.49	171	2.60	1.04 (0.89–1.23)	0.601	1.03 (0.86–1.23)^a^	0.751
							1.05 (0.87–1.26)^b^	0.644
Preterm birth (<37^+0^ weeks)	21 196	61.02	3695	56.27	0.93 (0.90–0.96)	<0.001		
Extremely preterm (<28^+0^ weeks)	936	44.16	173	46.82				
Very preterm (28^+0^ to 31^+6^ weeks)	1912	90.21	336	90.93				
Moderately preterm (32^+0^ to 36^+6^ weeks)	18 348	865.64	3186	862.25				

^a^ Adjusted for maternal age, parity, body mass index.

^b^ Adjusted for maternal age, parity, body mass index, and smoking status.

**FIGURE 2:**
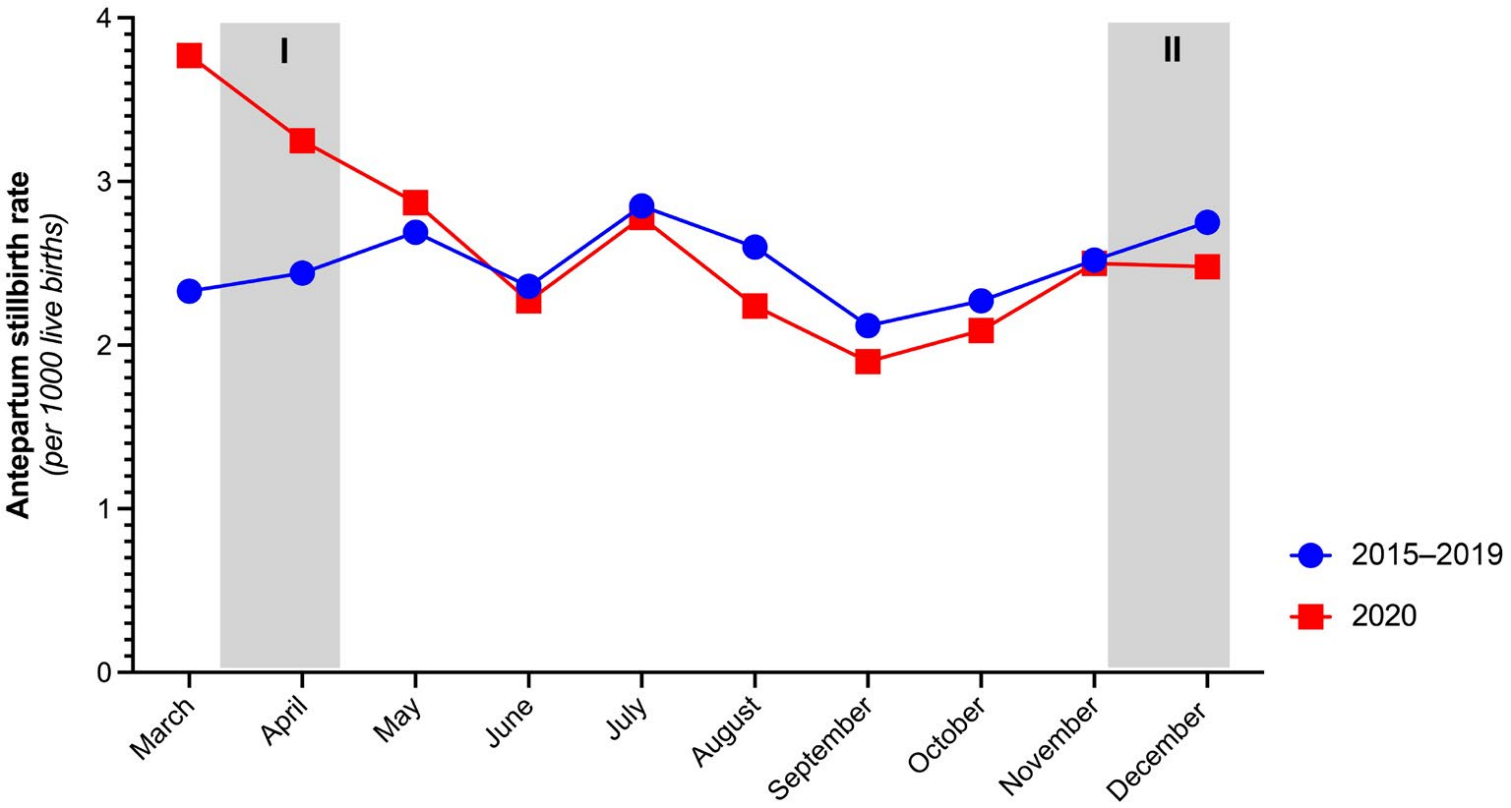
Monthly antepartum stillbirth rates per 1000 live births during the COVID‐19 pandemic (March to December 2020) and the preceding years (2015–2019) in Austria. Grey column signifies the first (I) lockdown (March 16 to April 13, 2020) and the second (II) lockdown (November 17 to December 6, 2020) in Austria

In particular, during the first lockdown phase, the risk of experiencing stillbirth increased to RR 1.48 (95% CI 1.06–1.06; *P *= 0.022), and to RR 1.56 (95% CI 1.08–2.27; *P *= 0.018) after adjustment for maternal age, BMI, and nicotine consumption (Table 2), resulting in an increased stillbirth rate from 2.38 to 3.52 per 1000 live births between March and April 2020, compared with the preceding years (*P *= 0.021; Figure 2).

**TABLE 2:** Risk for stillbirth and preterm birth in Austria during the lockdown period (March to April 2020) in comparison to the same time period of the preceding years

	Events (per 1000 births)				Odds ratio (95% confidence interval)			
	Pre‐COVID−19		COVID−19					
	Births in March to April 2015–2019		Births in March to April 2020		Unadjusted	*P* value	Adjusted	*P* value
	(*n* = 66 334)		(*n* = 12 517)					
	*n*	‰	*n*	‰				
Stillbirth	158	2.38	44	3.52	1.48 (1.06–2.07)	0.022	1.45 (1.00–2.10)^a^	0.05
							1.57 (1.08–2.27)^b^	0.018
Preterm birth (<37^+0^ weeks)	3985	60.07	716	57.2	1.00 (0.88–1.14)	0.213		
Extremely preterm (<28^+0^ weeks)	213	53.45	40	57.2				
Very preterm (28^+0^ to 31^+6^ weeks)	397	99.62	68	94.97				
Moderately preterm (32^+0^ to 36^+6^ weeks)	3375	846.93	608	849.16				

^a^ Adjusted for maternal age, parity, body mass index.

^b^ Adjusted for maternal age, parity, body mass index, and smoking status.

### 3.3 Risk factors for experiencing antepartum stillbirth in Austria

Table 3 shows the fetal and maternal characteristics in antepartum stillbirth events during the pandemic and pre‐pandemic eras.

**TABLE 3:** Fetomaternal characteristics of stillbirth events in Austria from March to December, and from March to April, respectively, during the pandemic period and the preceding years^a^

	Pandemic months March to December			Lockdown months March to April		
	Pre‐COVID−19 (2015–2019)	COVID−19 (2020)	*P* value	Pre‐COVID−19 (2015–2019)	COVID−19 (2020)	*P* value
Fetal						
Stillbirth events	866	171		158	44	
Gestational week	32.9 ± 5.0	33.7 ± 4.8	0.085^b^	32.5 ± 4.8	33.1 ± 4.9	0.426^b^
Sex						
Male	436 (50.3%)	95 (55.6%)	0.424^c^	84 (53.2%)	20 (45.5%)	0.556^c^
Female	429 (49.5%)	76 (44.4%)		73 (46.2%)	24 (54.5%)	
Unknown	1 (0.1%)			1 (0.6%)		
Weight (g)	1984 ± 989	2141 ± 1016	0.072^b^	1924.9 ± 1006.4	2026.2 ± 984.8	0.491^b^
Length (cm)	43.5 ± 7.5	44.7 ± 7.5	0.057^b^	43.0 ± 7.4	43.6 ± 7.5	0.578^b^
Maternal						
Age (years)	30.6 ± 5.9	31.0 ± 5.5	0.581^b^	30.8 ± 6.0	30.5 ± 5.0	0.918^b^
Parity						
Nullipara	418 (48.3%)	90 (52.6%)	0.219^c^	70 (44.3%)	25 (56.8%)	0.311^c^
Primipara	227 (26.2%)	34 (19.9%)		46 (29.1%)	11 (25.0%)	
Multipara	221 (25.5%)	47 (27.5%)		42 (26.6%)	8 (18.2%)	
Body Mass Index (kg/m^2^)	24.5 ± 4.7	25.0 ± 6.1	0.882^b^	24.7 ± 4.3	23.4 ± 5.3	0.036^b^
WHO BMI Classification						
Underweight	36 (5.1%)	10 (7.0%)	0.007^c^	4 (3.1%)	3 (8.3%)	0.167^c^
Normal weight	394 (55.9%)	83 (58.0%)		74 (57.8%)	26 (72.2%)	
Pre‐obesity	190 (27.0%)	22 (15.4%)		35 (27.3%)	4 (11.1%)	
Obesity I	62 (8.8%)	16 (11.2%)		12 9.4%)	2 (5.6%)	
Obesity II	15 (2.1%)	7 (4.9%)		2 (1.6%)		
Obesity III	8 (1.1%)	5 (3.5%)		1 (0.8%)	1 (2.8%)	
Nicotine	88 (10.2%)	8 (4.9%)	0.032^c^	20 (12.7%)	3 (6.8%)	0.276^c^
Previous fetal loss	2 (0.2%)	3 (1.8%)	0.009^c^		1 (2.3%)	0.057^c^
Gestational diabetes	25 (2.9%)	9 (5.3%)	0.111^c^	2 (1.3%)	2 (4.5%)	0.167^c^
Other pregnancy risks	22 (2.5%)	5 (33.3%)	<0.001^c^	4 (2.5%)	15 (34.1%)	<0.001^c^

Abbreviations: BMI, body mass Index (calculated as weight in kilograms divided by the square of height in meters); COVID‐19, coronavirus disease 2019; WHO, World Health Organization.

^a^ Values are presented as number, mean ± standard deviation, or as number (percentage).

^b^ Unpaired *t* test with level of significance *P* < 0.05.

^c^ χ^2^ test with level of significance *P* < 0.05.

The event of antepartum stillbirth during the pandemic was strongly related to increased fetal weight (RR 1.00, 95% CI 1.00–1.00; *P* < 0.001) and obesity class III (i.e., BMI ≥ 40.0: RR 2.54, 95% CI 1.00–6.40; *P* = 0.049), whereas previous fetal loss (RR 2.31, 95% CI 0.94–5.69; *P* = 0.069), other obstetric risk factors (RR 0.72, 95% CI 0.51–1.03; *P* = 0.072), obesity class II (i.e., 35.0–39.0: RR 1.69, 95% CI 0.76–3.74; *P* = 0.200), smoking (RR 1.33, 95% CI 0.65–2.71; *P* = 0.432), nulliparity (OR 1.01, 95% CI 0.69–1.49; *P* = 0.95), and preterm deliveries (OR 0.77, 95% CI 0.47–1.26; *P* = 0.305) were non‐influential.

## 4 DISCUSSION

During the COVID‐19 pandemic in Austria, overall obstetric characteristics had become significantly unfavorable in terms of concomitant pregnancy risk factors, advanced maternal age, and both maternal and fetal weight. Throughout the pandemic starting from March 2020, the Austrian stillbirth rate increased slightly by 1.1 cases per 100 live births, yet, during the first lockdown phase from March to April 2020, the stillbirth rate had significantly increased by 11.4 cases per 100 live births, culminating in the highest rate since the implementation of the Austrian Birth Registry, surpassing the peak of 3.05‰ in the year 2011. In identifying epidemiological characteristics for stillbirths in Austria during the pandemic, the adverse perinatal outcome was strongly associated with maternal and fetal weight, yet women experiencing stillbirth were found to more frequently have other obstetric risk factors and to more frequently have had previous fetal losses.

Our findings suggest that the nationwide lockdown with rapid disruption of healthcare services had a profound effect on the general well‐being of pregnant women and the surveillance of their pregnancies. The changes in the Austrian healthcare system might have also decreased potential numbers of iatrogenic prematurity to avoid adverse perinatal outcome, especially in the case of severely restricted fetuses, but the increase in stillbirth rates seems to act as a testimony for possible preventable fetal losses, if there had been sufficient opportunities to adequately monitor and manage high‐risk pregnancies, which had been state of the art during the pre‐COVID era.

The worldwide restrictions aimed at preventing COVID‐19 transmission seem to have exacerbated antenatal health across Europe, although, to date, the data are controversial. Based on a case series from the UK Obstetric Surveillance System study noting three stillbirths in 265 women with SARS‐CoV‐2 infection,^7^ the incidence of stillbirth among women affected by COVID‐19 has been extrapolated to 11.5 per 1000 total births, reflecting a three‐fold increase compared with the national rate of 4.1 per 1000 live births in the UK.^8^ As a reaction to gather more robust evidence, institutional, regional and national data have been analyzed. In a large London‐based tertiary referral center, the comparison of antepartum stillbirths (above 22 weeks of gestation and excluding terminations of pregnancy) from October 2019 to January 2020 and February to June 2020 resulted in a four‐fold institutional increase in the local stillbirth rate from 1.19‰ to 6.98‰ (OR 5.79, 95% CI 1.54–10.1; *P *= 0.01),^9^ with a constantly high incidence of 14.2‰ throughout the following months.^10^ To counterbalance these local data, a further UK study assessed regional and national hospitalization data from England, including antepartum stillbirths above 24 weeks of gestation, during the pandemic from April to June 2020 and in the pre‐pandemic period between 2016 and 2019, concluding neither a regional nor a national increase in stillbirth rates in England.^11^ These data support the findings of another institutional study from the UK showing no statistical difference in rates of stillbirths or decrease in preterm births.^12^ Further data published on stillbirth rates in Europe continued to reflect the conflicting evidence. A regional study from Italy observed a three‐fold increase from 1.07‰ to 3.23‰ between March and May 2020 versus 2019.^13^ In contrast, in Denmark, stillbirth rates declined from a pre‐pandemic rate of 3.3‰ to 2.7‰ during the lockdown from April to May 2020 (adjusted OR 0.78, 95% CI 0.57–1.06) with no compensatory increase in the rate of preterm delivery.^14^ Likewise, Spanish data confirm no increase either (OR 0.9, 95% CI 0.37–2.18)^15^ as do the population data from Germany, showing a pre‐pandemic stillbirth rate of 4.24‰ between January and July 2019 compared with 4.15‰ between the respective months during the COVID‐19 pandemic.^16^

Recent meta‐analyses on population stillbirth rates during the pandemic and historical cohorts from the pre‐pandemic era show that, in high‐income countries, stillbirth rates, unrelated to maternal SARS‐CoV‐2 infection, remained stable, as shown in the synthesis of 21 studies from 18 countries (OR 1.08, 95% CI 0.94–1.23; *P *= 1.23; adjusted OR 1.06, 95% CI 0.81–1.38); in a further 14 studies from nine countries (OR 1.38, 95% CI 0.94–2.02; *P* = 0.099; *I^2^* = 52%)^18^; or another analysis including 12 studies (OR 1.113, 95% CI 0.834–1.485).^19^ In accordance with our findings, an increase in birth weight was reported by six studies (mean difference 17 g, 95% CI 7–28 g) during the pandemic period.^17^

However, we agree that the incidence of antepartum stillbirth is still alarmingly high, and that “there has never been a more urgent time” to conceal the direct and indirect drivers of increased stillbirth rates during the pandemic.^20^

The major strength of our study is the high quality of its validated data derived from a nationwide birth registry. Our strict inclusion and exclusion criteria resulted in a homogeneous and concise cohort of antepartum fetal deaths only, excluding late terminations of pregnancy and fetal congenital malformations, which may have led to stillbirth irrespective of the pandemic. Furthermore, the present study design comparing two distinct time periods, reduced potential seasonal variances that could influence the overall stillbirth rates.

Our study is limited by its retrospective design with potentially missing data, errors in maternal data collection across study sites, and the failure to control for recall bias. Of note, we lack insight into the exact causes of fetal death in individual cases, and also, no data were available on the maternal SARS‐CoV‐2 infection status before, during, and after the event of stillbirth. However, for the latter, the low numbers of pregnant women in Austria who had COVID‐19 during the study period, and particularly during the first lockdown phase, need to be considered.

In conclusion, in Austria, there has been an overall rise in the antepartum stillbirth rate during the COVID‐19 pandemic. Particularly during the first lockdown phase, there has been a significant incidence peak, associated with maternal and fetal weight. This should be considered with regard to future perinatal management, in order to maintain high‐quality perinatal care during the ongoing COVID‐19 pandemic.

## CONFLICTS OF INTEREST

The authors have no conflicts of interest.

## AUTHOR CONTRIBUTIONS

We confirm that all authors fulfilled all conditions required for authorship, as all authors contributed to the conception, planning, and carrying out of the research. DAM and AF conceived the study. DAM wrote the first draft of this paper. DAM, KW, HK, and AF designed the study. SN, HH, and HL acquired the data. DAM, SN, and HL conducted the statistical analyses. All authors contributed to critically revising the paper for important intellectual content and approved the final version to be published. All authors accept responsibility for the article as published.

## References

[1] World Health Organization: WHO's COVID‐19 response [Online]. Available: http://www.who.int/emergencies/diseases/novel‐coronavirus‐2019/interactive‐timeline.

[2] Favre G , Pomar L , Qi X , Nielsen‐Saines K , Musso D , Baud D . Guidelines for pregnant women with suspected SARS‐CoV‐2 infection. Lancet Infect Dis. 2020;20:652‐653. 32142639 10.1016/S1473-3099(20)30157-2 PMC7134390

[3] Palmrich P , Roessler B , Wisgrill L , et al. Multiprofessional perinatal care in a pregnant patient with acute respiratory distress syndrome due to COVID‐19. BMC Pregnancy Childbirth, 2021;21:587. 34445988 10.1186/s12884-021-04059-y PMC8390084

[4] Wong YP , Khong TY , Tan GC . The effects of COVID‐19 on placenta and pregnancy: what do we know so far? Diagnostics. 2021;11(1):94. 33435547 10.3390/diagnostics11010094 PMC7827584

[5] Hillyard M , Sinclair M , Murphy M , Casson K , Mulligan C . The impact of COVID‐19 on the physical activity and sedentary behaviour levels of pregnant women with gestational diabetes. PLoS One. 2021;16:e0254364. 34415931 10.1371/journal.pone.0254364 PMC8378749

[6] Goyal M , Singh P , Singh K , Shekhar S , Agrawal N , Misra S . The effect of the COVID‐19 pandemic on maternal health due to delay in seeking health care: experience from a tertiary center. Int J Gynaecol Obstet. 2021;152:231‐235. 33128794 10.1002/ijgo.13457 PMC9087665

[7] Knight M , Bunch K , Vousden N , et al. Characteristics and outcomes of pregnant women admitted to hospital with confirmed SARS‐CoV‐2 infection in UK: national population based cohort study. BMJ. 2020;369:m2107. 32513659 10.1136/bmj.m2107 PMC7277610

[8] Magee LA , Khalil A , von Dadelszen P . Covid‐19: UK Obstetric Surveillance System (UKOSS) study in context. BMJ. 2020;370:m2915. 32718946 10.1136/bmj.m2915

[9] Khalil A , von Dadelszen P , Draycott T , Ugwumadu A , O'Brien P , Magee L . Change in the incidence of stillbirth and preterm delivery during the COVID‐19 pandemic. JAMA. 2020;324:705‐706. 10.1001/jama.2020.12746 PMC7435343 32648892

[10] Khalil A , von Dadelszen P , Ugwumadu A , Draycott T , Magee LA . Effect of COVID‐19 on maternal and neonatal services. Lancet Glob Health. 2021;9:e112. 33227255 10.1016/S2214-109X(20)30483-6 PMC7833114

[11] Stowe J , Smith H , Thurland K , Ramsay ME , Andrews N , Ladhani SN . Stillbirths during the COVID‐19 pandemic in England, April‐June 2020. JAMA. 2021;325:86‐87. 33284314 10.1001/jama.2020.21369 PMC7786243

[12] Vimalesvaran S , Shetty M , Khashu M . Letter to the Editor on the original article "Lack of changes in preterm delivery and stillbirths during COVID‐19 lockdown in a European region" by Juan Arnaez. Eur J Pediatr. 2021;1‐2. 10.1007/s00431-021-04071-6 PMC8039499 33844050

[13] De Curtis M , Villani L , Polo A . Increase of stillbirth and decrease of late preterm infants during the COVID‐19 pandemic lockdown. Arch Dis Child Fetal Neonatal Ed. 2021;106(4):456. 33127736 10.1136/archdischild-2020-320682 PMC8237197

[14] Pasternak B , Neovius M , Söderling J , et al. Preterm birth and stillbirth during the COVID‐19 pandemic in sweden: a nationwide cohort study. Ann Intern Med. 2021;174(6):873‐875. 33428442 10.7326/M20-6367 PMC7808327

[15] Arnaez J , Ochoa‐Sangrador C , Caserío S , et al. Lack of changes in preterm delivery and stillbirths during COVID‐19 lockdown in a European region. Eur J Pediatr. 2021;180(6):1997‐2002. doi:10.1007/s00431-021-03984-6 33580293 PMC7880019

[16] Kniffka MS , Nitsche N , Rau R , Kühn M . Stillbirths in Germany: on the rise, but no additional increases during the first COVID‐19 lockdown. Int J Gynaecol Obstet. 2021. doi:10.1002/ijgo.13832 PMC9087793 34287881

[17] Yang J , D’Souza R , Kharrat A , et al. COVID‐19 pandemic and population‐level pregnancy and neonatal outcomes: a living systematic review and meta‐analysis. Acta Obstet Gynecol Scand. 2021;100(10):1756‐1770. doi:10.1111/aogs.14206 34096034 PMC8222877

[18] Chmielewska B , Barratt I , Townsend R , et al. Effects of the COVID‐19 pandemic on maternal and perinatal outcomes: a systematic review and meta‐analysis. Lancet Glob Health. 2021;9(6):e759‐e772. 33811827 10.1016/S2214-109X(21)00079-6 PMC8012052

[19] Mohan M , Appiah‐Sakyi K , Oliparambil A , Pullattayil AK , Lindow SW & Konje JC . A meta‐analysis of global stillbirth rates during the COVID‐19 pandemic. Authorea June 23, 2021. doi:10.22541/au.162441694.43272789/v1 PMC10707675 38068270

[20] Homer CSE , Leisher SH , Aggarwal N , et al. Counting stillbirths and COVID 19‐there has never been a more urgent time. Lancet Glob Health. 2021;9:e10‐e11. 33212029 10.1016/S2214-109X(20)30456-3 PMC10011432

